# Characterization of Mesenchymal and Neural Stem Cells Response to Bipolar Microsecond Electric Pulses Stimulation

**DOI:** 10.3390/ijms26010147

**Published:** 2024-12-27

**Authors:** Giorgia Innamorati, Marina Sanchez-Petidier, Giulia Bergafora, Camilla Codazzi, Valentina Palma, Francesca Camera, Caterina Merla, Franck M. André, Maria Pedraza, Victoria Moreno Manzano, Laura Caramazza, Micol Colella, Paolo Marracino, Marco Balucani, Francesca Apollonio, Micaela Liberti, Claudia Consales

**Affiliations:** 1 PhD Program in Cellular and Molecular Biology, Department of Biology, University of Rome “Tor Vergata”, 00133 Rome, Italy; 2 Division of Biotechnologies, Italian National Agency for Energy, New Technologies and Sustainable Economic Development (ENEA), 00123 Rome, Italy; giulia.bergafora@gmail.com (G.B.); camillacodazzi@gmail.com (C.C.); valentina.palma@enea.it (V.P.); francesca.camera@enea.it (F.C.); caterina.merla@enea.it (C.M.); 3 Neural Circuits and Behaviour Laboratory, Fundación Hospital Nacional de Parapléjicos, 45004 Toledo, Spain; marinas@externas.sescam.jccm.es; 4 Metabolic and Systemic Aspects of the Oncogenesis (METSY), CNRS, Institut Gustave Roussy, Université Paris-Saclay, 94805 Villejuif, France; franck.andre@cnrs.fr; 5 Neuronal and Tissue Regeneration Laboratory, Centro de Investigación Príncipe Felipe (CIPF), 46012 Valencia, Spain; pedrazaboti1@gmail.com (M.P.); vmorenom@cipf.es (V.M.M.); 6 BioEMLab Group, DIET, Department of Information Engineering, Electronics and Telecommunications Sapienza, University of Rome, 00184 Rome, Italy; laura.caramazza@uniroma1.it (L.C.); micol.colella@uniroma1.it (M.C.); francesca.apollonio@uniroma1.it (F.A.); micaela.liberti@uniroma1.it (M.L.); 7 Rise Technology Srl, 00121 Rome, Italy; paolo.marracino@risetechnology.com (P.M.); marco.balucani@risetechnology.com (M.B.)

**Keywords:** stem cells, microsecond electric pulse stimulation, cell proliferation, gene expression

## Abstract

In the tissue regeneration field, stem cell transplantation represents a promising therapeutic strategy. To favor their implantation, proliferation and differentiation need to be controlled. Several studies have demonstrated that stem cell fate can be controlled by applying continuous electric field stimulation. This study aims to characterize the effect of a specific microsecond electric pulse stimulation (bipolar pulses of 100 µs + 100 µs, delivered for 30 min at an intensity of 250 V/cm) to induce an increase in cell proliferation on mesenchymal stem cells (MSCs) and induced neural stem cells (iNSCs). The effect was evaluated in terms of (i) cell counting, (ii) cell cycle, (iii) gene expression, and (iv) apoptosis. The results show that 24 h after the stimulation, cell proliferation, cell cycle, and apoptosis are not affected, but variation in the expression of specific genes involved in these processes is observed. These results led us to investigate cell proliferation until 72 h from the stimulation, observing an increase in the iNSCs number at this time point. The main outcome of this study is that the microsecond electric pulses can modulate stem cell proliferation.

## 1. Introduction

Stem cells are undifferentiated or partially differentiated cells of different origins (embryonic and adult) that can differentiate into multiple cell types. Thanks to this feature, they represent a very promising therapeutic strategy for all those diseases characterized by permanently damaged and/or degenerated tissue. In the nervous system, for instance, neuronal stem cells can differentiate into neural lineage. Indeed, lines of evidence demonstrate that when transplanted, these cells can give rise to neurons and/or glial cells, which, secreting cytokines and growth factors, can sustain tissue repair by inhibiting cell apoptosis and fibrosis [1,2,3].

To effectively use stem cells in tissue regeneration, it is very important to be able to control their fate by directing their differentiation into the desired cell type. However, one of the major obstacles during transplantation is that a significant portion of the implanted cells die during the engraftment process. For this reason, promoting their proliferation once they reach the tissue to be regenerated could help mitigate the issue of poor engraftment.

One of the methods used to manipulate stem cell fate is to stimulate with an electrical current [4,5]. Lines of evidence have demonstrated how electrical stimulation applied in different current modes, such as alternating, monophasic, biphasic, and continuous, as well as with different frequency, voltage, and duration ranges, allows to determine different biological responses in different cell types, stem cells included. Current studies, indeed, show that externally applied electrical stimulation can impact neural stem cell morphology, migration, proliferation, apoptosis, and differentiation [6,7,8,9].

All living cells present natural electric fields. Bioelectricity arises from a different accumulation of electric charges across cell membranes which is kept at its homeostasis by the passive flux of ions, or their active movement through ion channels [8]. This phenomenon highlights that the cells have a stable transmembrane potential (TMP), which makes them sensitive to the application of an external electric field that can alter this balance of charges. The response of the cells to this type of stimulation depends on the intensity, the method of application, and the duration, as well as the type of cells analyzed [9].

Ultrashort electric pulses (PEFs), lasting from nano to milliseconds, are delivered at high intensity and can charge the cell membrane, creating large localized electric fields across it. When the charging goes beyond a threshold, small pores (electroporation) on the cell membrane are formed, and depending on the intensity, this process can be, or not, be reversible [10,11,12]. This means that by adjusting the applied intensities, it is possible to induce reversible or irreversible electroporation [13]. The pore formation allows direct access into the cell of molecules that usually cannot cross the plasma membrane. For example, Ca^2+^, can only enter the cytoplasm, or be released from the endoplasmic reticulum, through channel proteins. Applying PEFs, this ion can diffuse from the extracellular medium into the cytosol; furthermore, if the electric amplitude and the pulse duration are high enough, Ca^2+^ can also be released from the inner stores [11,14].

Calcium is one of the most important second messengers, regulating the main processes of cellular homeostasis. In stem cells, it plays a pivotal role in determining cellular fate [15]. In the nervous system, its function is essential, especially in the process of neurogenesis, where sequential bursts of Ca^2+^ induce neuronal stem/progenitor cells to migrate, proliferate, and differentiate into neurons and glia [16,17,18].

The possibility of controlling stem cell fate, acting on their calcium fluxes, through bipolar microsecond electric pulse stimulation is the starting point of the RISEUP FET-OPEN project (grant agreement n. 964562). The main topic of this project is to develop an innovative approach to treat spinal cord injuries by transplanting MSCs, derived from adipose tissue, and iNSCs, obtained from induced pluripotent stem cells, and stimulating them, by an electrified biocompatible scaffold, with microsecond pulses to firstly induce their proliferation, and then their neuronal and/or neuronal-like differentiation by modulating their intracellular calcium oscillation. In particular, by increasing the number of calcium oscillations, cells enhance their proliferation; conversely, by blocking them, cells differentiate [19,20].

In this manuscript, we present the biological response induced in adipose tissue-derived MSCs and iNSCs, derived from iPSC differentiation, in response to 100 µs + 100 µs bipolar pulses, at an intensity of 250 V/cm, delivered for 30 min every 30 s. This protocol induces a boost in calcium oscillation (CNRS manuscript in preparation), so we analyzed the effect on cell homeostasis, by evaluating cell proliferation, cell cycle, and apoptosis, and on the expression of the genes specifically involved in these processes to verify if this protocol could effectively induce stem cells proliferation.

## 2. Results

### 2.1. Lack of Effect of µsPEFs on Cell Growth, Cell Cycle, and Apoptosis 24 Hours After the Stimulation

**Figure 1:**
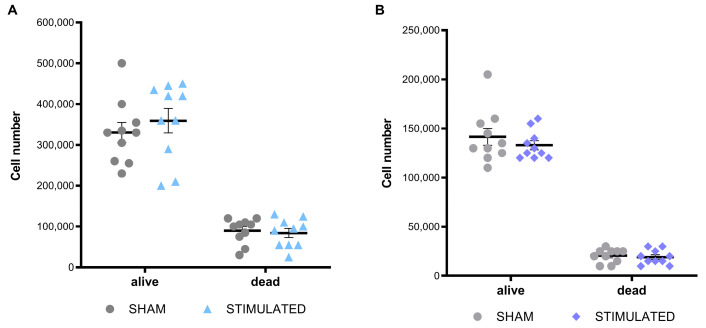
No significant effect of µsPEFs on cell number. Number of (**A**) iNSCs and (**B**) MSCs assessed through cell counting 24 h after the µsPEFs stimulation, comparing the number of stimulated cells with the sham. Mean values ± SEM are shown (n = 10). Statistics performed by unpaired Two-tailed t-test. Significant differences were recognized as a *p*-value < 0.05 and indicated with asterisks as follows: * *p* < 0.05; ** *p* < 0.01; *** *p* < 0.001.

Cell growth, cell cycle, and the apoptosis of iNSCs and MSCs were evaluated 24 h after the stimulation to characterize the general effect of µsPEFs on these cells’ viability. At the end of each experiment, the cells were counted considering both alive and dead cells. Comparing the results obtained from the stimulated cells and the sham, no statistically significant variations in the cell number were observed either in iNSCs (Figure 1A) or in MSCs (Figure 1B).

A deep investigation of this result was performed by evaluating the cell cycle through propidium iodide staining, and apoptosis, considering the cleavage of the caspase-3 through Western blot analysis.

**Figure 2:**
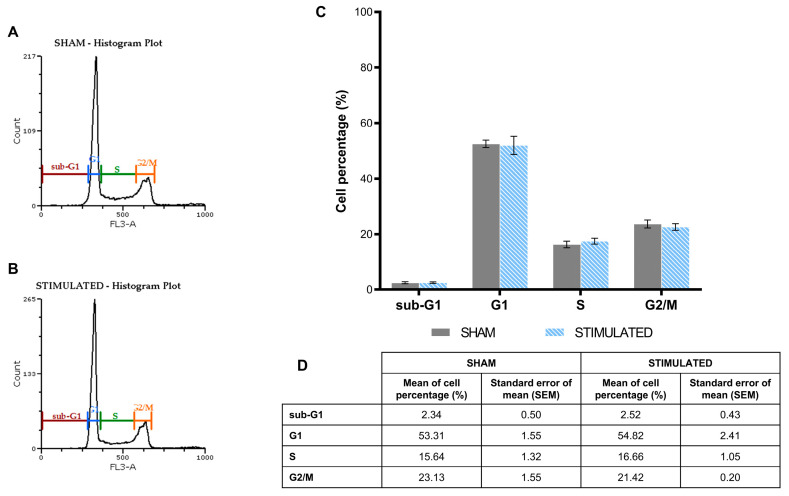
No significant effect of µsPEFs on iNSC cell cycle. Cell cycle analysis of iNSCs performed through propidium iodide (PI)/RNase staining buffer 24 h after the µsPEFs stimulation, comparing the stimulated cells with the sham. (**A**) Histogram example of the cell cycle analysis of the sham. (**B**) Histogram example of the cell cycle analysis of the stimulated cells. (**C**) Bar graph summarizing the effect of µsPEFs stimulation on cell cycle. (**D**) Mean values ± SEM are shown (n = 3). Statistics performed by unpaired Two-tailed t-test. Significant differences were recognized as a *p*-value < 0.05 and indicated with asterisks as follows: * *p* < 0.05; ** *p* < 0.01; *** *p* < 0.001.

**Figure 3:**
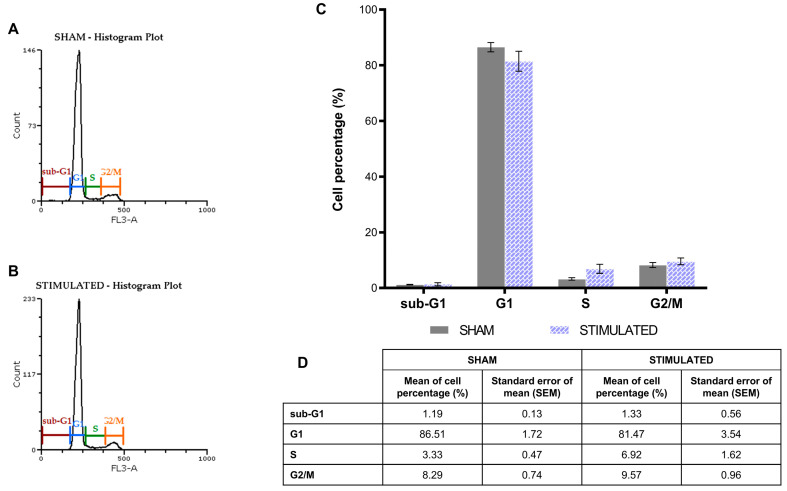
No significant effect of µsPEFs on MSCs cell cycle. Cell cycle analysis of MSCs performed through propidium iodide (PI)/RNase staining buffer 24 h after the µsPEFs stimulation, comparing the stimulated cells with the sham. (**A**) Histogram example of the cell cycle analysis of the sham. (**B**) Histogram example of the cell cycle analysis of the stimulated cells. (**C**) Bar graph summarizing the effect of µsPEFs stimulation on cell cycle. (**D**) Mean values ± SEM are shown (n = 3). Statistics performed by unpaired Two-tailed t-test. Significant differences were recognized as a *p*-value < 0.05 and indicated with asterisks as follows: * *p* < 0.05; ** *p* < 0.01; *** *p* < 0.001.

The phases of the iNSCs (Figure 2) and MSCs (Figure 3) cell cycle were not affected by the stimulation; the chosen protocol does not induce variations in cell cycle, proliferation (no changes on G1 phase) and mortality (no changes on sub-G1 phase) compared to the sham. Details about the performed analysis are shown in Figures S1 (iNSCs) and S2 (MSCs).

**Figure 4:**
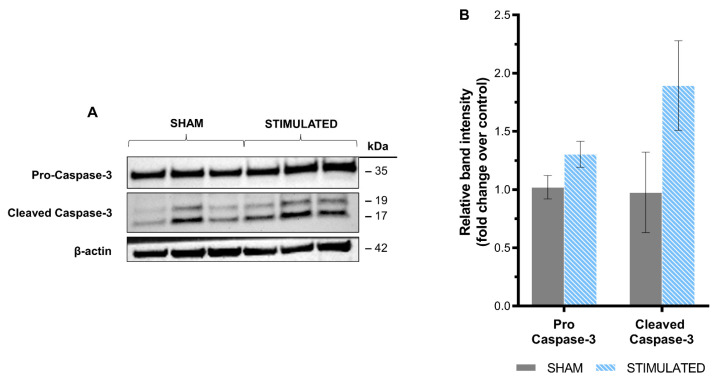
No significant increase in Caspase-3 level in iNSCs after 24 h from the stimulation with µsPEFs. Apoptosis in iNSCs was evaluated by assessing variation in Caspase-3 cleavage through the Western blot analysis, comparing the stimulated cells with sham. (**A**) The Western blot analysis of Caspase-3 cleavage showing the protein level of pro-Caspase-3 (35 kDa), cleaved Caspase-3 (19–17 kDa), and β-actin (42 kDa) as the loading control. (**B**) Bar graph summarizing the variation in protein level after densitometric analysis, comparing the stimulated cells with the sham. Mean values ± SEM are shown (n = 3). Statistics performed by unpaired Two-tailed *t*-test. Significant differences were recognized as a *p*-value < 0.05 and indicated with asterisks as follows: * *p* < 0.05; ** *p* < 0.01; *** *p* < 0.001.

**Figure 5:**
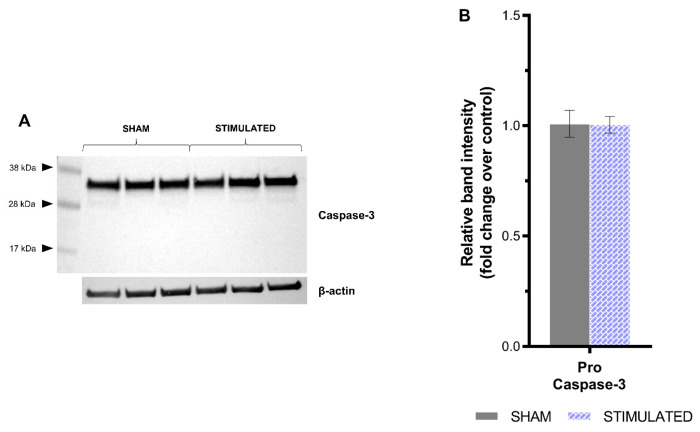
No significant increase in Caspase-3 level in MSCs after 24 h from the stimulation with µsPEFs. Apoptosis in MSCs was evaluated by assessing variation in Caspase-3 cleavage through the Western blot analysis, comparing the stimulated cells with sham. (**A**) The Western blot analysis of Caspase-3 cleavage showing the protein level of pro-Caspase-3 (35 kDa), cleaved Caspase-3 (19–17 kDa), and β-actin (42 kDa) as the loading control. (**B**) Bar graph summarizing the variation in protein level after densitometric analysis, comparing the stimulated cells with the sham. Mean values ± SEM are shown (n = 3). Statistics performed by unpaired Two-tailed t-test. Significant differences were recognized as a *p*-value < 0.05 and indicated with asterisks as follows: * *p* < 0.05; ** *p* < 0.01; *** *p* < 0.001.

The results deriving from the apoptosis evaluation confirmed the previous highlights: no activation of apoptosis through Caspase-3 cleavage was observed in the stimulated cells compared with the sham, neither in iNSCs (Figure 4) nor in MSCs (Figure 5). In detail, in iNSCs, pro-Caspase-3 (35 kDa) and cleaved Caspase-3 (19–17 kDa) were detected by the Western blot analysis (Figure 4A), but no statistically significant differences were observed after image analysis compared to the sham (Figure 4B). Instead, in MSCs, only pro-Caspase-3 (35 kDa) was detected (Figure 5A) without statistically significant variations resulting from the µsPEF stimulation (Figure 5B). The absence of cleaved Caspase-3 shows that the stimulation protocol chosen does not activate the enzyme and the associated biochemical and morphological changes. Positive control obtained through etoposide treatment is shown in Figures S3 and S4.

These results point out that at 24 h, a clear effect on cell proliferation is not visible, but also demonstrate that this protocol of stimulation is not toxic for iNSCs and MSCs, because no cell death is induced.

### 2.2. Gene Expression

#### 2.2.1. µsPEFs Affect Immediate Early Genes (IEGs) Expression

**Figure 6:**
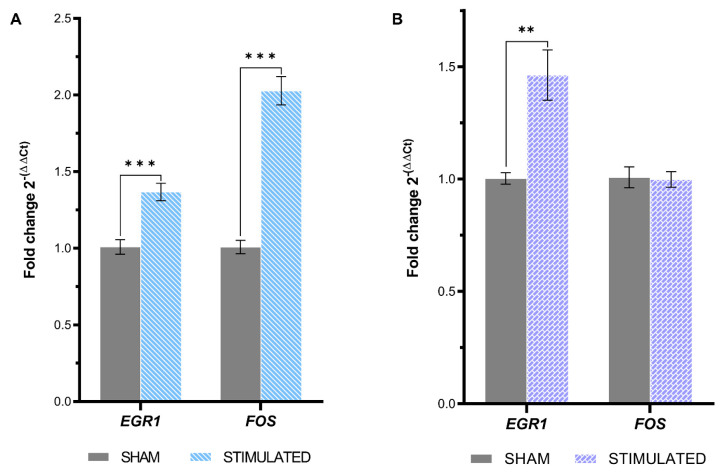
IEGs expression resulting from µsPEFs stimulation. IEGs expression evaluated through qRT-PCR 1 h after the µsPEFs stimulation. (**A**) Bar graph representing the IEGs expression in iNSCs. (**B**) Bar graph representing the IEGs expression in MSCs. Mean values ± SEM are shown (n = 9). Statistics performed by unpaired Two-tailed *t*-test. Significant differences were recognized as a *p*-value < 0.05 and indicated with asterisks as follows: * *p* < 0.05; ** *p* < 0.01; *** *p* < 0.001.

To assess the immediate molecular response of iNSCs and MSCs to the selected µsPEFs stimulation protocol, the expression of two immediate early genes (IEGs), *EGR1* (Early Growth Response 1) and *FOS* (Fos Proto-Oncogene), was evaluated 1 h after the stimulation. As shown in Figure 6A, the expression of both IEGs was statistically significant increase with a 1.37-fold for *EGR1* (*p*-value < 0.001) and a 2.03-fold for *FOS* (*p*-value < 0.001) in the stimulated iNSCs compared to the sham. However, in MSCs, only the expression of *EGR1* was raised by the stimulation with a 1.46-fold (*p*-value = 0.003) (Figure 6B).

#### 2.2.2. Different Effects of µsPEFs on Genes Involved in Proliferation, Cell Cycle, and Apoptosis

As shown in Section 2.1, no significant effects on cell growth, cell cycle, and apoptosis were observed 24 h after the µsPEFs stimulation. Despite this, a deep investigation of the expression of genes involved in these biological processes was carried out both in iNSCs and MSCs. In detail, the expression of *MKI67* (encoding for Ki-67) and *MYC* (encoding for c-Myc) was assessed as markers of cells proliferation, *CCND1* (encoding for cyclinD1), *CCNDE1* (encoding for cyclinE1), and *E2F1* (encoding for the transcriptional factor E2f1) as genes involved in cell cycle, and *BAX* (encoding for the pro-apoptotic protein Bax) and *BCL2* (encoding for the anti-apoptosis protein Bcl2) to highlight effects on apoptosis.

**Figure 7:**
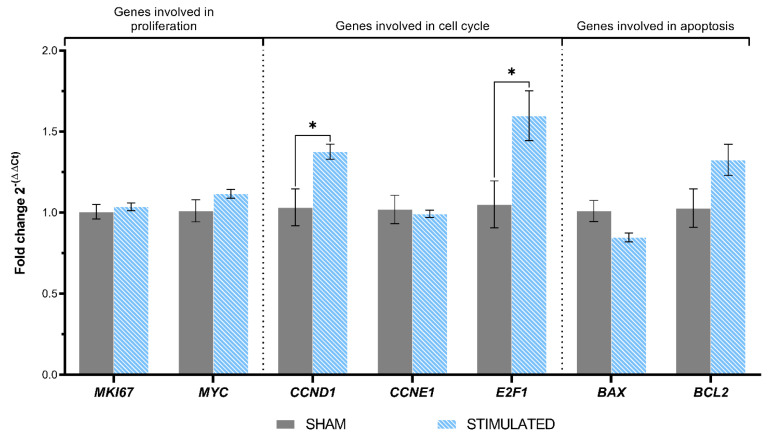
Gene expression resulting from the µsPEFs stimulation of iNSCs. The expression of the genes involved in proliferation, cell cycle, and apoptosis evaluated through qRT-PCR in iNSCs 24 h after the stimulation, comparing the stimulated cells with the sham. Mean values ± SEM are shown (n = 9). Statistics performed by unpaired Two-tailed *t*-test. Significant differences were recognized as a *p*-value < 0.05 and indicated with asterisks as follows: * *p* < 0.05; ** *p* < 0.01; *** *p* < 0.001.

After 24 h, the stimulation induces on iNSCs a statistically significant increase in *CCND1* and *E2F1* expression, encoding for the proteins involved in the cell cycle G1/S transition, with a 1.38-fold (*p*-value = 0.027) and a 1.59-fold (*p*-value = 0.027), respectively. Instead, the other genes analyzed were not affected by the stimulation protocol chosen (details shown in Figure 7).

**Figure 8:**
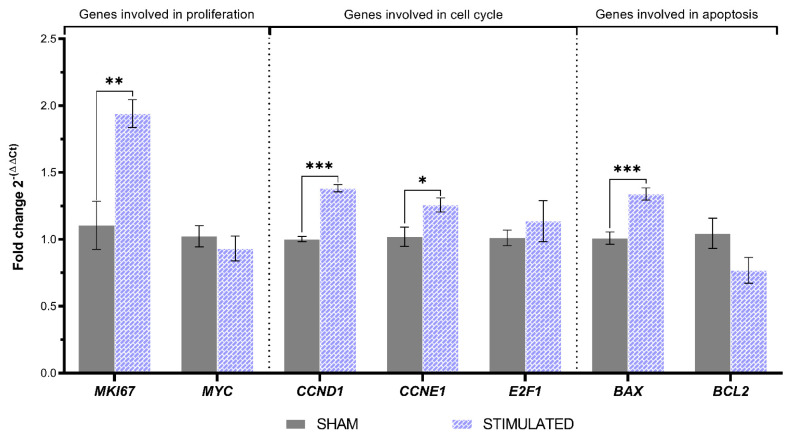
Gene expression resulting from the µsPEFs stimulation of MSCs. The expression of the genes involved in proliferation, cell cycle, and apoptosis evaluated through qRT-PCR in MSCs 24 h after the stimulation, comparing the stimulated cells with the sham. Mean values ± SEM are shown (n = 9). Statistics performed by unpaired Two-tailed *t*-test. Significant differences were recognized as a *p*-value < 0.05 and indicated with asterisks as follows: * *p* < 0.05; ** *p* < 0.01; *** *p* < 0.001.

About MSCs, the expression of several considered genes was affected by the stimulation. Indeed, a statistically significant increase in the expression of the proliferative marker *MKI67* was observed (1.94-fold and *p*-value = 0.001), also associated with a rise in the expression of *CCND1* and *CCNE1,* involved in the cell cycle G1/S transition, with a 1.38-fold (*p*-value < 0.001) and a 1.26-fold (*p*-value = 0.02), respectively. Regarding the genes involved in apoptosis, µsPEFs induced an increased expression of *BAX*, involved in the apoptotic pathway (1.34-fold and *p*-value < 0.001). All the details about the gene expression of MSCs are shown in Figure 8.

#### 2.2.3. Genes Involved in Stemness Are Affected by µsPEFs

In the context of stem cells, another interesting aspect that could be affected by the µsPEFs is stemness. *SOX2* (SRY-Box Transcription Factor 2) and *POU5F1* (POU Class 5 Homebox 1) expressions were analyzed to assess variations in pluripotency, proliferative potential, and self-renewal capacity 24 h after the stimulation.

**Figure 9:**
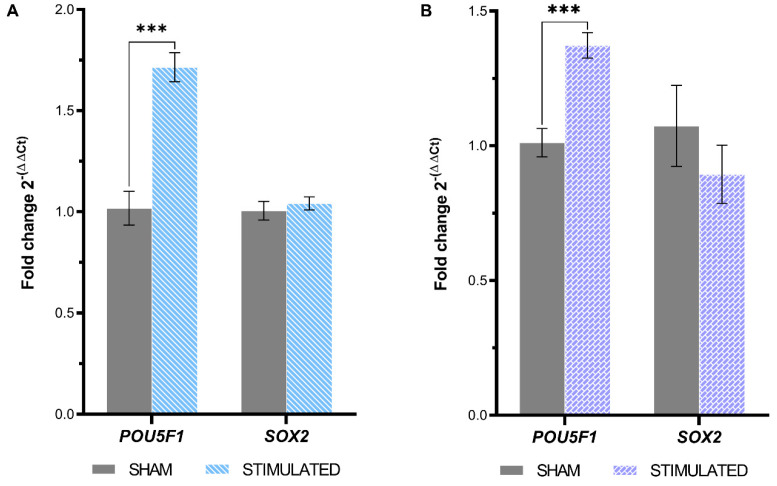
Expression of genes involved in stemness resulting from µsPEFs stimulation. The expression of the genes involved in stemness evaluated through qRT-PCR in (**A**) iNSCs and (**B**) MSCs 24 h after the stimulation, comparing the stimulated cells with the sham. Mean values ± SEM are shown (n = 9). Statistics performed by unpaired Two-tailed *t*-test. Significant differences were recognized as a *p*-value < 0.05 and indicated with asterisks as follows: * *p* < 0.05; ** *p* < 0.01; *** *p* < 0.001.

The results, shown in Figure 9, highlight a statistically significant increase in the expression of *POU5F1* compared to the sham in both iNSCs (1.75-fold and *p*-value < 0.001) (Figure 9A) and MSCs (1.37-fold and *p*-value < 0.001) (Figure 9B).

### 2.3. µsPEFs Have Different Effects on Cell Growth

The results described above, especially those regarding gene expression and IEGs, suggest an effect of µsPEFs on cell proliferation, not visible after 24 h. For this reason, this parameter was evaluated in both cell lines after 24 h, 48 h, and 72 h from the stimulation, by cell counting and the analysis of Ki-67 levels (proliferation marker).

**Figure 10:**
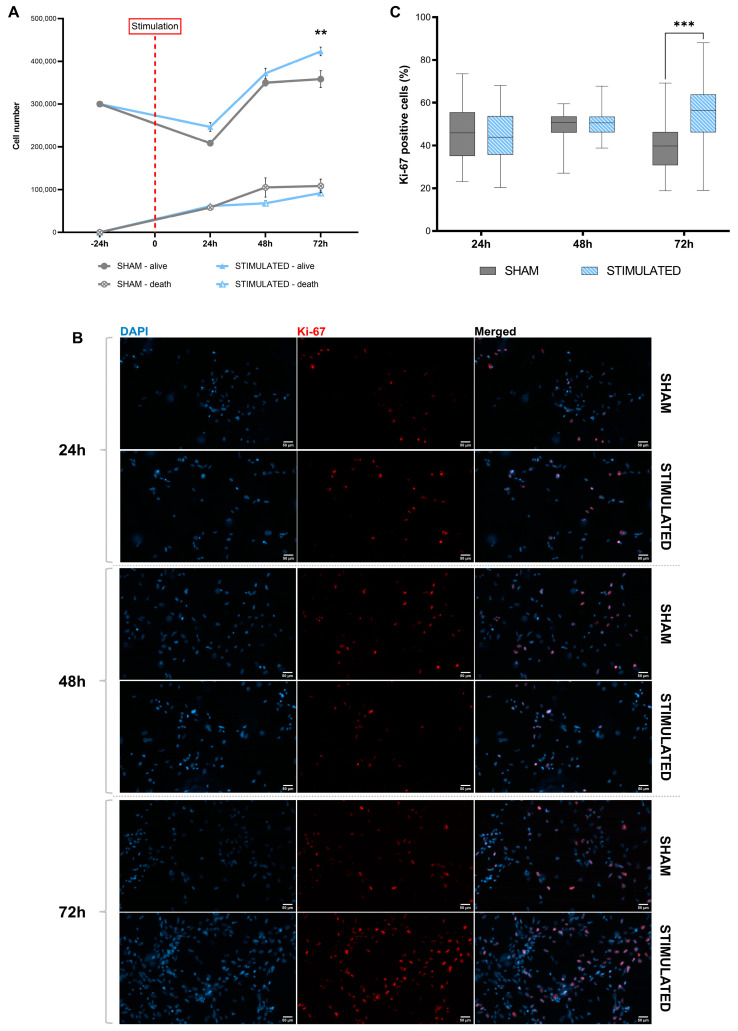
Analysis of iNSCs proliferation, after 24 h, 48 h, and 72 h from the stimulation. (**A**) Growth curve obtained by counting the stimulated cells and sham at different time points. Mean values ± SEM are shown (n = 3). (**B**) Representative images of Ki-67 staining performed on iNSCs with and without the stimulation at different time points. Cell labeled with Ki-67 (red) and DAPI (cyan). Scale bars: 50 μm. (**C**) Percentage of Ki-67-positive cells. Statistics were performed by two-way ANOVA followed by post hoc analysis with the Sidak test. Significant differences were recognized as a value *p* < 0.05 and indicated with asterisks as follows: * *p* < 0.05; ** *p* < 0.01; ** *p* < 0.001.

In iNSCs, after 72 h, the stimulation induced an increase in the number of cells compared to the sham (*p*-value = 0.002) (Figure 10A). The data were confirmed by immunofluorescence analysis, aimed at identifying the percentage of cells positive to Ki-67 (red in Figure 10B); an increase in this positivity was observed after 72 h compared to the sham (Figure 10C) (*p*-value < 0.001).

**Figure 11:**
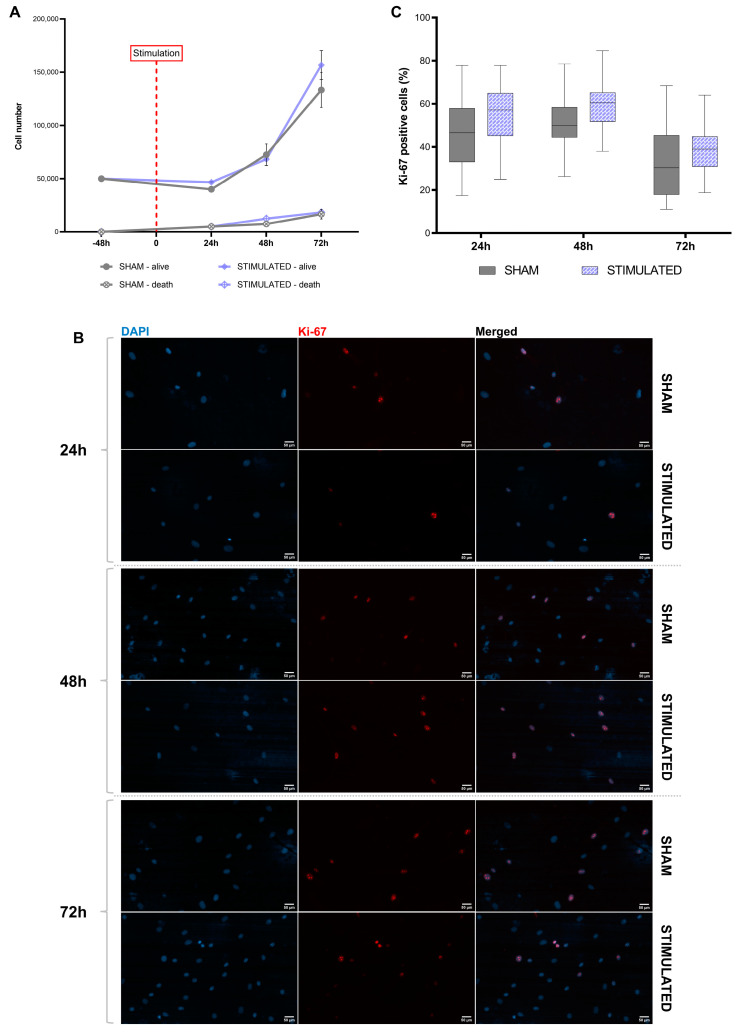
Analysis of MSCs proliferation, after 24 h, 48 h, and 72 h from the stimulation. (**A**) Growth curve obtained by counting the stimulated cells and sham at different time points. Mean values ± SEM are shown (n = 3). (**B**) Representative images of Ki-67 staining performed on iNSCs with and without the stimulation at different time points. Cell labeled with Ki-67 (red) and DAPI (cyan). Scale bars: 50 μm. (**C**) Percentage of Ki-67-positive cells. Statistics were performed by two-way ANOVA followed by post hoc analysis with the Sidak test. Significant differences were recognized as a value *p* < 0.05 and indicated with asterisks as follows: * *p* < 0.05; ** *p* < 0.01; *** *p* < 0.001.

In MSCs, this effect was not observed; indeed, no statistically significant changes were observed in the cell number even though an increasing trend was present (Figure 11A). These data were confirmed by the analysis of Ki-67-positive cells; indeed, no variations were detected (Figure 11B,C).

## 3. Discussion

Stem cells represent a very powerful tool for the regeneration of damaged tissues. Since their discovery, several studies have focused on their use in various degenerative pathologies and for tissue regeneration, for instance in the context of spinal cord injuries.

The success of cell transplantation depends on several factors: the ability of the cells to colonize the injured tissue, their ability to survive, and their ability to differentiate. For this reason, it is very important to apply different strategies to achieve these goals. In addition to transplantation using scaffolds to promote engraftment, it is also necessary to apply protocols that can favor initial cell proliferation (to improve their survival) and, subsequently, their differentiation.

In this manuscript, we investigated the possibility of inducing the proliferation of two types of stem cells, human MSCs and iNSCs, using ultrashort electrical pulses in the microsecond range. These pulses are widely used in biology and in clinical practice due to their ability to interact with the cell membrane, inducing the formation of pores without causing temperature alterations. This allows their use to transfer genetic material and/or drugs inside the cells (electrochemotherapy), or to induce irreversible electroporation, used in anticancer therapy [21,22].

We applied microsecond pulses with an energy density around the electroporation threshold, able to modulate intracellular calcium fluxes, which is another effect induced by this type of stimulation [11]. In particular, the protocol applied, bipolar 100 µs + 100 µs, 250 V/cm, can induce an increase in the intracellular calcium oscillation (CNRS manuscript in preparation), and the hypothesis is that this effect can enhance cell proliferation. Being the first time that such kind of protocol of stimulation was applied to iNSCs and MSCs, even the first time ever that iNSCs were stimulated with microsecond electric pulses, we decided to initially evaluate the response of these cells 24 h after the stimulation, also to determine whether these pulses were safe for cells.

By assessing cell homeostasis through live cell count, cell cycle, and apoptosis analysis, we found that there was no increase in proliferation, no change in cell cycle phases, and no cell death was induced. Therefore, this stimulation protocol was absolutely safe for the two types of stem cells considered and did not affect cell homeostasis 24 h after the stimulation, but we could not observe any influence on cell growth, which was the main goal we wanted to obtain with this protocol.

Therefore, we focused on trying to understand the type of molecular response induced. We initially analyzed the expression of two immediate early genes, *EGR1* and *FOS*. These genes encode two transcription factors that have a very short activation time and are immediately inactivated after their function. There are many conditions that induce their transcription and transduction because their function is to activate the molecular response of the cell to external stimuli [23]. They are the playmakers of the cell proliferation induction [24,25,26]. Furthermore, both these genes are involved in the processes of neurogenesis and signal transmission in the central nervous system [27,28,29].

We have already demonstrated, with different stimulation protocols, that ultrashort electric pulses, both microsecond and nanosecond, can induce the transcription of these IEGs [12]; also in this case, the 100 µs + 100 µs bipolar pulses, at an intensity of 250 V/cm protocol is able to enhance the expression of both genes in iNSCs, while of only *EGR1* in MSCs. *EGR1* is downstream of calcium signaling, so its induction in both cell types suggests that the observed calcium fluxes could be responsible for the increase in its expression, which, in turn, was suggestive of a possible role in increasing cell proliferation.

Subsequently, analyzing the expression of the genes specifically involved in the process of proliferation, cell cycle, and apoptosis, we observed a modulation of some of them in both cell types. Furthermore, both iNSCs and MSCs showed a statistically significant increase in the *POU5F1* gene. This gene encodes the transcription factor Oct4 which possesses a POU domain and plays a fundamental role in embryonic development and stem cell pluripotency [30]. This result seems to confirm that effectively, both MSCs and iNSCs, under the pulses protocol stimulation described before, maintain their stemness characteristics, and the increase in POU5F1 expression is in line with an effect on cell proliferation. Therefore, considering that at a “gross” level 24 h after stimulation we did not see any effect on proliferation and cell cycle, but at a molecular level the expression of genes involved in these processes were modulated, we thought that above all, for proliferation, 24 h could not be sufficient time to observe a variation. We decided to repeat the experiment by stimulating the cells and then counting them up to the next 72 h. We, thus, observed that iNSCs showed a statistically significant increase in proliferation, while MSCs did not, even if they still showed an increasing trend. These results demonstrated that stimulating stem cells with 100 µs + 100 µs bipolar pulses, at an intensity of 250 V/cm protocol, effectively induces an enhancement in iNSCs growth. This result was also confirmed by evaluating the Ki-67 level. This protein increases its expression as the cells progress through the cell cycle with the highest level in G2/M and lowest in quiescent or resting G0 cells, which makes it a powerful marker of cell proliferation [31]. The lack of significance observed in MSCs, even though 24 h after the stimulation an enhanced expression of the Ki-67 gene is observable, could be due to the different duplication times of these cells which are lower than iNSCs. Furthermore, it is worth noting that as for any other treatment applied to the cells, the effects induced by the electric stimulation can be cell-specific. The cellular response to electrical stimulation depends on the transmembrane potential which is dynamic and changes with metabolism, cell cycles, and states of differentiation. This is applicable both to differentiated cells such as neurons, which, for example, at rest stage are hyperpolarized, and to stem cells, which usually result in depolarization. Also, among similar cells, the effect of electrical stimulation can induce different responses. iPSC and ESCs, while being electrophysiologically stable, show different levels of expression of genes coding for ion channels [32,33]. On the contrary, MSCs, differently from these types of cells, are extremely heterogeneous from the electrophysiological point of view, which is also reflected in their metabolism and differentiation capacity [34].

As a different response to the electric stimulation of the two cell types, we observed only in MSCs a statistically significant increase in *BAX* expression, which codifies for a protein promoting cell apoptosis, even though no apoptosis induction was visible with Western blot analysis. Cell apoptosis is induced when BAX is enhanced and BCL-2, an anti-apoptotic protein, is decreased [35]. In our experiments, *BCL-2* expression is not affected, and, furthermore, the fold change in *BAX* increase is not so high, which would explain the apparent inconsistency of our results.

Several studies have demonstrated that neuronal differentiation, both of MSCs and iNSCs, can be achieved by electrical stimulation [6,36,37,38]; moreover, it is possible to influence the neural lineage of differentiated iNSCs through electrical stimulation [8]. Responsible for the response to electrical stimulation are the voltage-gated calcium channels [37,39]. These allow the entry of extracellular calcium following stimulation, which then acts as a second messenger internally, activating signal transduction and regulating a series of essential functions [40,41,42]. Also in our work, we hypothesize that the calcium pathway, activated by the microsecond electric pulse stimulation, could be involved in the observed effects. The different duplication times and the involvement of voltage-dependent channels would also explain the different responses of the two cell lines. In fact, iNSCs have a greater number of channels, which makes them much more sensitive to stimulation than MSCs [37,43,44]. Experiments to define the transduction pathway activated are currently underway.

## 4. Materials and Methods

### 4.1. Cell Culture

MSCs, adipose tissue-derived, were obtained from Dr. F. André, CNRS, through the signing of a Material Transfer Agreement (MTA). iNSCs were from Dr. Moreno Manzano, CIPF, after the signing of a MTA.

Human MSCs were maintained in complete Dulbecco’s modified Eagles medium (DMEM) with high glucose concentration (4500 mg/L), completed with 10% heat-inactivated fetal bovine serum, 100 U/mL of L-glutamine, 100 U/mL penicillin and 100 U/mL streptomycin.

Human iNSCs were maintained in STEMdiff Neural Progenitor Basal Medium completed with Supplement A (50X), Supplement B (1000X) (STEMCELL Technologies, Vancouver, BC, Canada), 100 U/mL of penicillin, and 100 U/mL of streptomycin. To maintain their growth in adhesion, a coating of Geltrex^TM^ LDEV-Free (Gibco, Thermo Fisher Scientific Inc, Waltham, MA, USA) diluted 1:50 in DMEM/F-12 was used.

Both cell lines were cultured at 37 °C in a humidified incubator with 5% CO_2_ and used until passage 10.

### 4.2. Exposure System and Stimulation

**Figure 12:**
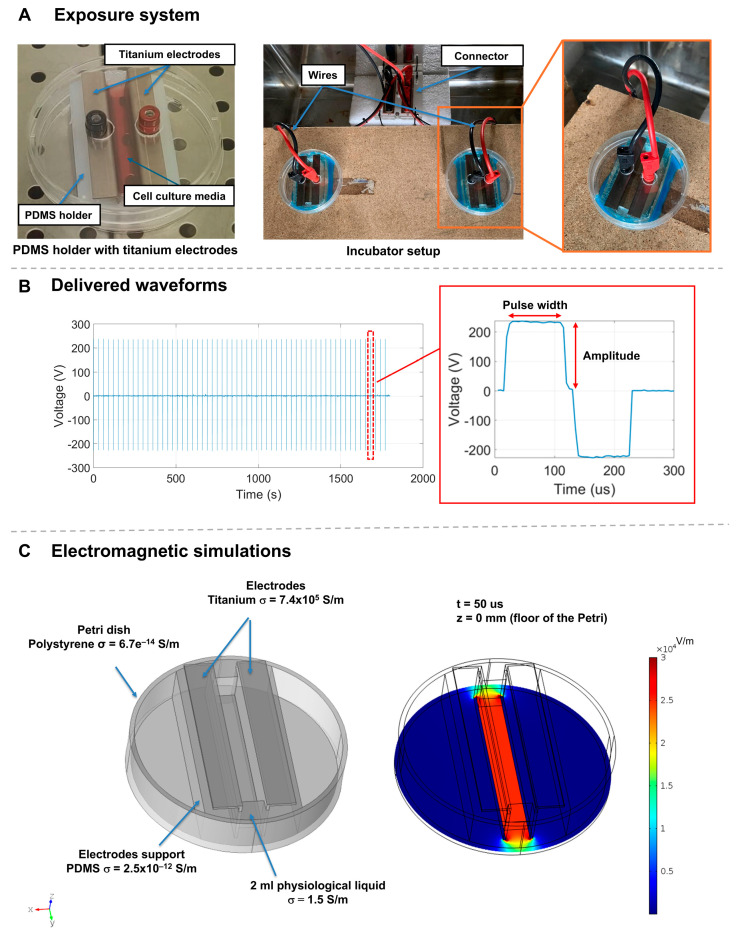
Representation of the exposure system used and the output signal describing the µsPEFs stimulation protocol. (**A**) PDMS holder was fabricated to maintain the titanium electrodes parallel and at 1 cm from each other. A picture of the incubator setup and a detail of the wire’s connection is shown. (**B**) Waveform delivered by the ELECTROcell B15 generator. (**C**) System model and electromagnetic stimulation result assessing the uniform exposure on the cells when the pulses are delivered.

The cells were exposed to microsecond electric pulses (µsPEFs) in 10 cm Petri dishes, where a pair of titanium electrodes were placed parallel at 1 cm from each other. To maintain the electrodes in this specific configuration, polydimethylsiloxane (PDMS) (The Dow Chemical Company, Midland, MI, USA) holders were fabricated. In addition, some modifications on the dish covers allowed the direct connection of the electrodes to the generator, keeping the Petri dishes closed for the entire stimulation time (Figure 12A).

The µsPEFs were delivered by the ELECTROcell B15 generator (LEROY Biotech, Saint-Orens-de-Gameville, France) previously connected to the titanium electrodes.

The system described was designed in CAD using COMSOL v5. After correctly assigning the materials, as depicted in Figure 12C, the system was simulated over time (5 μs steps) for a single bipolar pulse of the waveform (Figure 12B). The figure illustrates the electric field obtained at the bottom of the Petri dish (where the cells adhere) when the positive pulse has reached its plateau (at t = 50 μs, refer to Figure 12B). The field in the region where the cells are present is uniform at 250 V/cm.

For all the biological experiments, except growth curve and Ki-67 staining, 3.5 × 10^5^ iNSCs and 1.5 × 10^5^ MSCs were seeded in the exposure systems in a final volume of 3 mL of fresh medium. After 24 h for iNSCs and 48 h for MSCs from the seeding, the media was changed, and 2 mL of fresh medium was added; this volume is necessary to have the right current passing through the electrodes.

**Figure 13:**
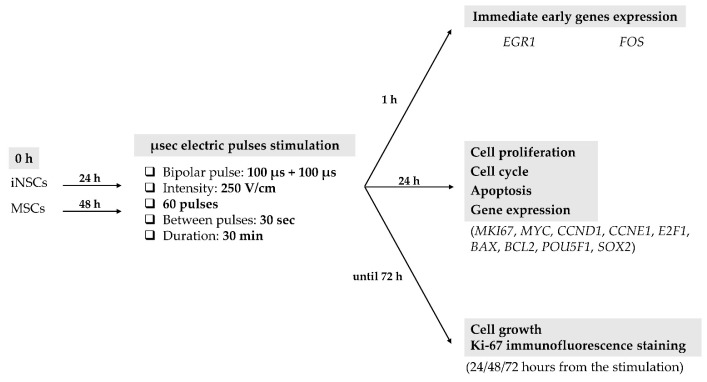
Schematic representation of the experimental protocol followed.

The cells were stimulated with microsecond electric bipolar pulses of 100 µs + 100 µs, and 2 pulses/minute were delivered for 30 min at an intensity of 250 V/cm. At the end, 1 mL of fresh medium was added to each exposure system, and the cells were maintained in culture until the biological experiments. The stimulations were performed in a properly modified incubator to guarantee the maintenance of the specific culture conditions during the entire time of the experiment. Based on the specific biological experiment to be carried out, the cells were harvested at different time points after the stimulation (the experimental protocol is described in detail in Figure 13).

For each experiment, sham samples were prepared plating the cells in the same conditions and in the same exposure system (PDMS and titanium electrodes), but no electric stimulation was applied.

### 4.3. Cell Growth Analysis

To assess the effect of the stimulation on the cell growth, the cells were counted after each experiment, comparing the stimulated one with the sham. Moreover, a growth curve was performed by counting the cells 24 h, 48 h, and 72 h from stimulation to assess a possible long-term effect. In this case, 3.0 × 10^5^ iNSCs and 5.0 × 10^4^ MSCs were seeded 24 h and 48 h before the stimulation, respectively.

For the counting, the cells were harvested, and the viability was assessed by Erythrosin B dye (Sigma-Adrich, St. Louis, MO, USA) exclusion.

### 4.4. Flow-Cytometric Analysis of Cell Cycle

After 24 h from the stimulation, the cells were harvested, washed with 1X PBS, and fixed with pre-cooled 70% ethanol. After overnight incubation at −20 °C, the cells were resuspended in propidium iodide (PI)/RNase staining buffer (λex = 490 nm, λem = 600 nm, BD Biosciences, Franklin Lakes, NJ, USA) and analyzed by flow cytometry using a FACSCalibur (BD Biosciences, Franklin Lakes, NJ, USA). A minimum of 10,000 events were evaluated. Data were acquired using the Cell Quest software (BD Biosciences, Franklin Lakes, NJ, USA) and analyzed using FCS Express version 7 (De Novo, Pasadena, CA, USA). Forward (FSC-H) and side scatterings (SSC-H) were used to exclude cellular debris from the analysis and to gate healthy cells.

### 4.5. Western Blotting for Apoptosis Evaluation

The cells harvested after 24 h from the stimulation were lysed in a Cell Extraction Buffer (Invitrogen, Carlsbad, CA, USA) supplemented with 1.0 mM phenylmethylsulfonyl fluoride and HaltTM protease and phosphatase inhibitor cocktail (100X) (both from Pierce Biotechnology, Rockford, IL, USA) on ice for 30 min. The lysates were centrifugated at 13,000 rpm for 10 min at 4 °C and the protein concentration in the supernatant was determined using the Coomassie Plus (Bradford) Assay Kit (Pierce Biotechnology, Rockford, IL, USA). An equal amount of protein for each sample was prepared to add 4X BoltTM LDS Sample Buffer and 10X BoltTM Reducing Agent (both from Invitrogen, Carlsbad, CA, USA) before loading onto wells of a pre-cast BoltTM Bis-Tris Plus Mini Protein Gels, 4–12%, 1.0 mm (Invitrogen, Carlsbad, CA, USA) and then transferred to nitrocellulose membranes (iBlot™ 2 Transfer Stacks, nitrocellulose, mini–Invitrogen, Carlsbad, CA, USA).

The membranes were incubated with blocking solution (TBS containing 0.05% Tween-20 and 5% nonfat dried milk powder) for 1 h at room temperature and then probed with the antibodies anti-Caspase-3 (Cell Signaling, Danvers, MA, USA, #9662; 1:1,000) and anti-β-actin (Sigma-Aldrich, St. Louis, MO, USA, #A5441, 1:5,000). Bands were detected after incubation with HRP-linked goat anti-rabbit (Cell Signaling, Danvers, MA, USA, #7074, 1:3,000) or anti-mouse (Cell Signaling, Danvers, MA, USA, #7076, 1:3,000) secondary antibodies through the ECL detection system (Life Technologies, Carlsbad, CA, USA) in combination with the iBrightTM FL1500 Imaging System (Invitrogen, Carlsbad, CA, USA). As a positive control for apoptosis detection, the cells were exposed for 5 h to etoposide (Sigma-Aldrich, St. Louis, MO, USA, Cas. n.: 33419-42-0) at the final concentrations of 5 µM and 10 µM and proteins were run into a gel with the same condition described for the samples. The quantification was performed using the ImageJ2 software version 1.52i [45].

### 4.6. Gene Expression Analysis

To evaluate the expression of several genes following the stimulation, total RNA was extracted at different time points: for the evaluation of the immediate early genes (IEGs) FOS and EGR1, the extraction was performed after 1 h from the stimulation instead, for all the other genes, after 24 h from the stimulation.

For the RNA extraction, the Direct-zol RNA Miniprep Kit (Zymo Research, Irvine, CA, USA) was used following the manufacturer’s instructions. The quantitative and qualitative evaluation of the extracted RNA was performed by Nanoready Touch F3100 (Hangzhou LifeReal Biotechnology Co., Zhejiang, China) calculating the 260/230 nm and 260/280 nm absorbance ratio. Five hundred nanograms of the total RNA were retrotranscribed into cDNA using the QuantiTect Reverse Transcription Kit (Qiagen, Venlo, The Netherlands). cDNA was then diluted 1:10 for real-time PCR analysis and amplified with specific primers using Luna^®^ Universal qPCR Master Mix (New England Biolabs, MA, USA).

The relative abundance of the specific mRNA levels was calculated by normalizing to GAPDH expression using the 2^−ΔΔCt^ methods and it was expressed as fold change. All the reactions were run in triplicate.

The complete list of the used primer sequences is shown in Table 1.

### 4.7. Immunofluorescence Assay

In each exposure system (shown in Figure 12A), 3 coverslips of 18 mm diameter were placed to completely cover the stimulation area before cell seeding. A total of 3.0 × 10^5^ iNSCs and 5.0 × 10^4^ MSCs were seeded, respectively, 24 h and 48 h before the stimulation.

After 24 h, 48 h, and 72 h from the stimulation, the cells were fixed with 4% paraformaldehyde (10 min at 37 °C), and permeabilized with 0.1% Triton X-100 in 1X PBS (15 min at room temperature). Blocking was performed in 2% BSA in 1X PBS (1 h at room temperature) and then the coverslips were probed with antibodies anti-Ki-67 (Invitrogen, Carlsbad, CA, USA, #MA5-14520; 1:500) in 0.1% BSA (overnight at 4 °C). Appropriate Alexa Fluor-conjugated secondary antibody (Invitrogen, Carlsbad, CA, USA, Goat anti-Rabbit IgG (H+L) Cross-Adsorbed Secondary Antibody, Alexa Fluor™ 594, #A-11012, 1:1,000) was used (45 min at room temperature). Finally, the samples were counterstained with DAPI (1:10,000 in 1X PBS) for 5 min at room temperature and mounted using Fluoroshield™ with DAPI (Sigma-Aldrich, St. Louis, MO, USA, # F60579). Images were captured (20 images per coverslip) with a Zeiss Axio Observer fluorescence microscope (Zeiss, Oberkochen, Germany). The images were analyzed and cellular nuclei were counted by the Analyze plugin of ImageJ2 software version 1.52i [45].

### 4.8. Statistical Analysis

Data were presented as mean ± standard error of the mean (SEM) calculated with n ≥ 3 replicates. All the graphical and statistical data analyses were performed using GraphPad Prism version 9 (GraphPad Software, San Diego, CA, USA). The *p*-values were determined using the Two-tailed t-test, except for the cell growth and the evaluation of Ki-67-positive cells where the significant differences in the values considered were measured by variance analysis (two-way ANOVA) followed by post hoc analysis with the Sidak test.

Significant differences were recognized as a *p*-value < 0.05 and indicated with asterisks as follows: * *p* < 0.05; ** *p* < 0.01; *** *p* < 0.001.

## 5. Conclusions

Bioelectricity and regeneration in multicellular organisms are deeply connected [46]. The electricity generated by cells can regulate morphogenesis in all living tissues. Organ regeneration involves the careful regulation of cell proliferation, movement, and positioning. During embryogenesis, electrical signals serve to direct the formation of organs and limbs [8]. Within the RISEUP project framework, we are developing microsecond stimulation protocols to control the proliferation and differentiation of iNSCs and MSCs to transplant them and achieve regeneration in spinal cord injury. This is a totally innovative application for µsPEFs since they are currently used for different aims and in different fields of application, such as gene and drug delivery [47], monoclonal antibody production [48], microbial decontamination [49], and to improve the overall quality of wine [50]. The advantages of using this kind of stimulation for cell proliferation, instead of the ones already described in the literature, principally based on direct current application, are that µsPEFs do not induce thermal effect, have very short timing of application, and are target-specific [51].

These first results described in the manuscript demonstrate that the protocol applied can enhance effectively iNSCs growth 72 h after the stimulation, while MSCs show a trend of increase. Further studies are necessary to analyze the molecular pathways activated, but certainly, these results open to the innovative and futuristic applications of microsecond electric pulses, different from those currently used in medical therapy.

## Figures and Tables

**Table 1:** Primers used for gene expression analysis through qRT-PCR.

Gene Name	Sequence
*EGR1*	Forward 5′-CAGCAGCCTTCGCTAACC-3′
	Reverse 5′-CCACTGGGCAAGCGTAA-3′
*FOS*	Forward 5′-TACTACCACTCACCCGCAGACT-3′
	Reverse 5′-GAATGAAGTTGGCACTGGAGAC-3′
*CCND1*	Forward 5′-TGTGCATCTACACCGACAACT-3′
	Reverse 5′-CACAGAGGGCAACGAAGGT-3′
*CCNE1*	Forward 5′-TGTGTCCTGGATGTTGACTGCC-3′
	Reverse 5′-CTCTATGTCGCACCACTGATACC-3′
*E2F1*	Forward 5′-GGACCTGGAAACTGACCATCAG-3′
	Reverse 5′-CAGTGAGGTCTCATAGCGTGAC-3′
*BAX*	Forward 5′-TCAGGATGCGTCCACCAAGAAG-3′
	Reverse 5′-TGTGTCCACGGCGGCAATCATC-3′
*BCL2*	Forward 5′-ATCGCCCTGTGGATGACTGAGT-3′
	Reverse 5′-GCCAGGAGAAATCAAACAGAGGC-3′
*MKI67*	Forward 5′-GAGGTGTGCAGAAAATCCAAA-3′
	Reverse 5′-CTGTCCCTATGACTTCTGGTTGT-3′
*MYC*	Forward 5′-CCTACCCTCTCAACGACAGC-3′
	Reverse 5′-CTCTGACCTTTTGCCAGGAG-3′
*POU5F1*	Forward 5′-CCTGAAGCAGAAGAGGATCACC-3′
	Reverse 5′-AAAGCGGCAGATGGTCGTTTGG-3′
*SOX2*	Forward 5′-GAGCTTTGCAGGAAGTTTGC-3′
	Reverse 5′-GCAAGAAGCCTCTCCTTGAA-3′
*GAPDH*	Forward 5′-GAGGGATCTCGCTCCTGGA-3′
	Reverse 5′-GCACCGTCAAGGCTGAGAAC-3′

## Data Availability

All the data generated during this work will be available at https://zenodo.org/communities/riseup/?page=1&size=20 (accessed on 12 December 2024).

## Supplementary Materials

The following supporting information can be downloaded at https://www.mdpi.com/article/10.3390/ijms26010147/s1.

## Abbreviations

**Abbreviations**	**Meaning**
µsPEFs	microsecond electric pulses
CIPF	Centro Investigación Príncipe Felipe
CNRS	Centre National de la Recherche Scientifique
DMEM	Dulbecco’s modified Eagles medium
EGR1	Early Growth Response 1
ESCs	Embryonic Stem Cells
FOS	Fos Proto-Oncogene
IEGs	immediate early genes
iNSCs	induced neural stem cells
iPSCs	induced pluripotent stem cells
MSCs	mesenchymal stem cells
MTA	Material Transfer Agreement
PDMS	polydimethylsiloxane
PEF	Pulsed electric field
PI	propidium iodide
POU5F1	POU Class 5 Homebox 1
RT	room temperature
SEM	standard error of the mean
SOX2	SRY-Box Transcription Factor 2
TMP	transmembrane potential
