# Supplementary material for: Characterization of Mesenchymal and Neural Stem Cells Response to Bipolar Microsecond Electric Pulses Stimulation
Source: Int J Mol Sci. 2024 Dec 27;26(1):147. doi: 10.3390/ijms26010147 (PMC11720446; doi:10.3390/ijms26010147)
Supplement: Supplementary file 1 [file ijms-26-00147-s001.zip › ijms-3284999-supplementary.pdf]

Supplementary Materials

Characterization of mesenchymal and neural stem cells response to bipolar microsecond pulses electric stimulation

Giorgia Innamorati[1,2], Marina Sanchez-Petidier[3], Giulia Bergafora[2], Camilla Codazzi[2], Valentina Palma[2], Francesca Camera[2], Caterina Merla[2], Maria Pedraza Boti[4], Victoria Moreno Manzano[4], Laura Caramazza[5], Micol Colella[5], Paolo Marracino[6], Marco Balucani[6], Franck M. André[3] and Claudia Consales[2*]

[1] PhD Program in Cellular and Molecular Biology, Department of Biology, University of Rome "Tor Vergata", Rome, Italy.; giorgia.innamorati@alumni.uniroma2.eu

[2] Division of Health Protection Technologies, Italian National Agency for Energy, New Technologies and Sustainable Economic Development (ENEA), 00123 Rome Italy; claudia.consales@enea.it, bergafora.1696601@studenti.uniroma1.it, codazzi.1852498@studenti.uniroma1.it, valentina.palma@enea.it, francesca.camera@enea.it, caterina.merla@enea.it.

[3] Neural Circuits and Behaviour Lab, Fundación Hospital Nacional de Parapléjicos, Toledo, Spain; marinas@externas.sescam.jccm.es

[4] CNRS, Metabolic and Systemic Aspects of the Oncogenesis, (METSY), Université Paris-Saclay, InstitutGustave Roussy, 94805 Villejuif, France; franck.andre@cnrs.fr

[5] Neuronal and Tissue Regeneration Laboratory, Centro de Investigación Príncipe Felipe (CIPF), Valencia, Spain; pedrazaboti1@gmail.com, vmorenom@cipf.es.

[6] BioEMLab Group, DIET, Department of Information Engineering, Electronics and Telecommunications Sapienza, University of Rome, Italy; laura.caramazza@uniroma1.it, micol.colella@uniroma1.it

[7] RISE TECHNOLOGY SRL, Ostia (RM), Italy; paolo.marracino@risetechnology.com

* Correspondence: claudia.consales@enea.it; Tel.: +390630484031

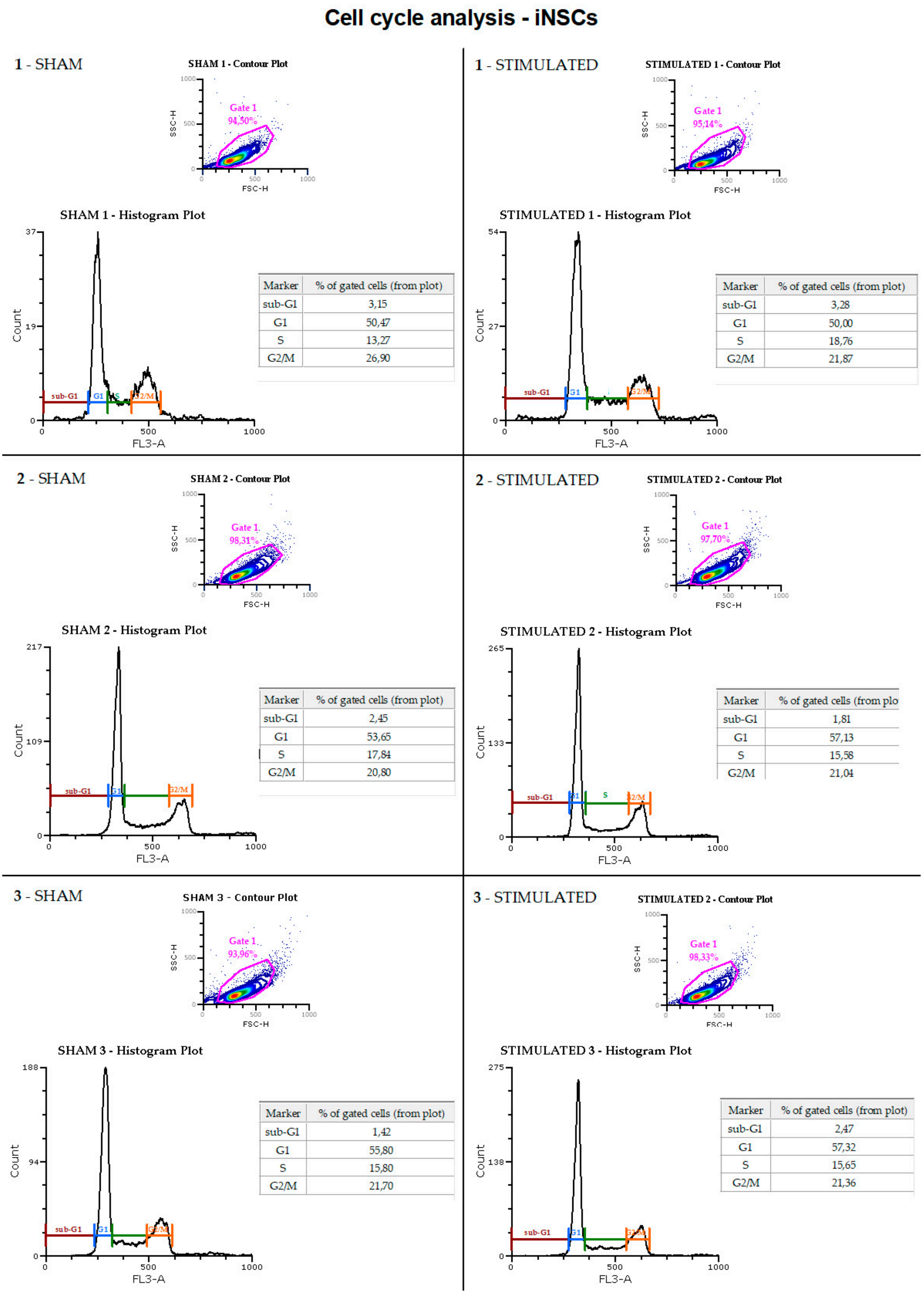

Figure S1. Details of the cell cycle analysis performed on iNSCs.
Detailed representation of the cell cycle analysis performed on iNSCs using FCS Express version 7. Contour and histogram plot are shown for all the samples analyzed, as well as the % of gated cells enclosed by each marker which highlight the different phases of the cell cycle.

Cell cycle analysis - MSCs

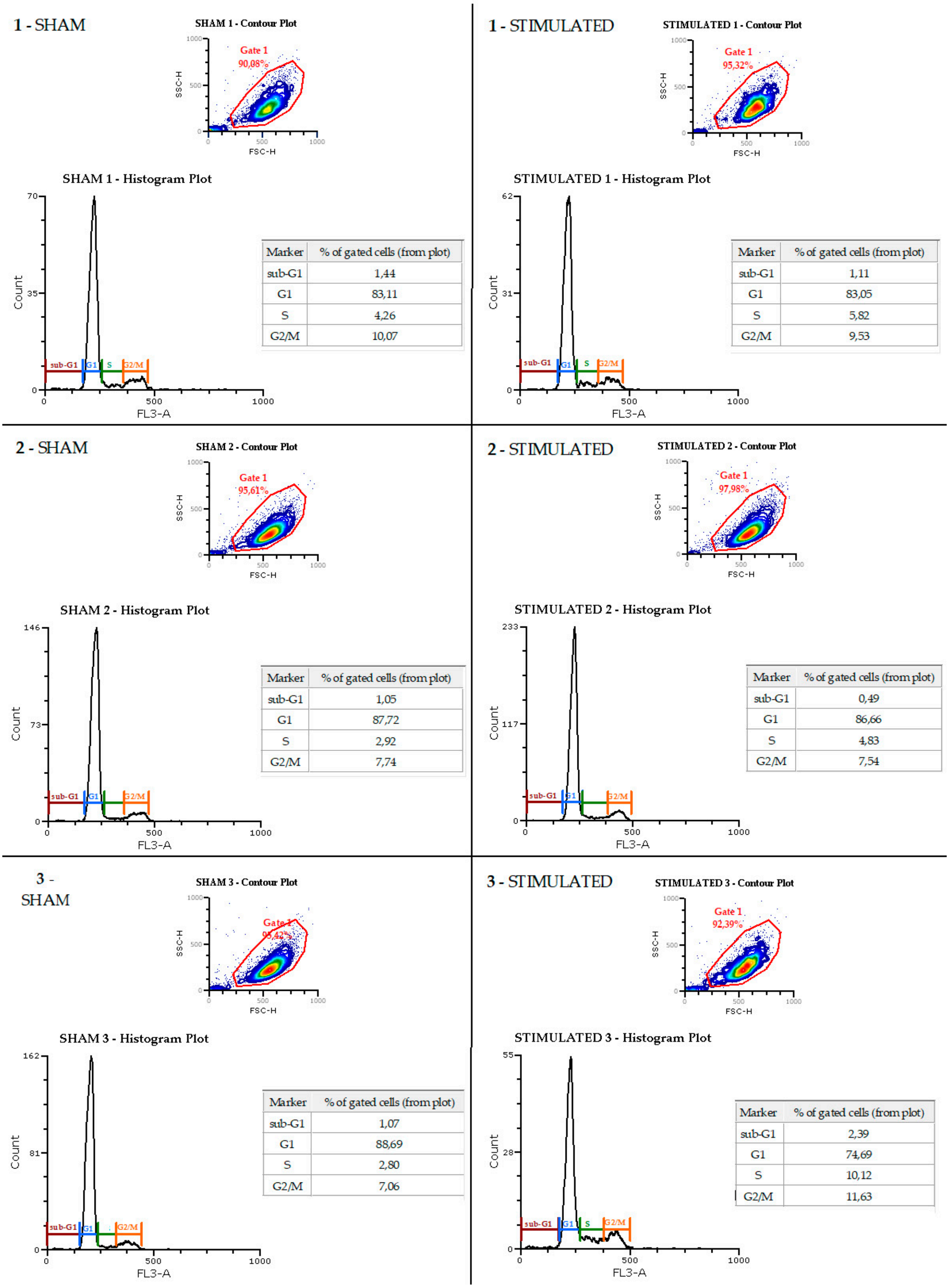

Figure S2. Details of the cell cycle analysis performed on MSCs.
Detailed representation of the cell cycle analysis performed on MSCs using FCS Express version 7. Contour and histogram plot are shown for all the samples analyzed, as well as the % of gated cells enclosed by each marker which highlight the different phases of the cell cycle.

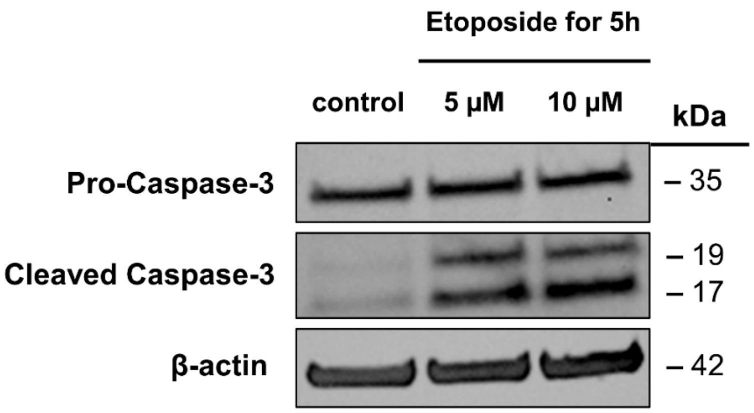

Figure S3. Apoptosis positive control obtained by treating iNSCs with final concentration of 5 µM and 10 µM of etoposide for 5h**.** Western blot showing protein level of Pro-Caspase-3 (35 kDa), Cleaved Caspase-3 (19-17 kDa) and β-actin (42 kDa), comparing the untreated control with the cells treated with etoposide.

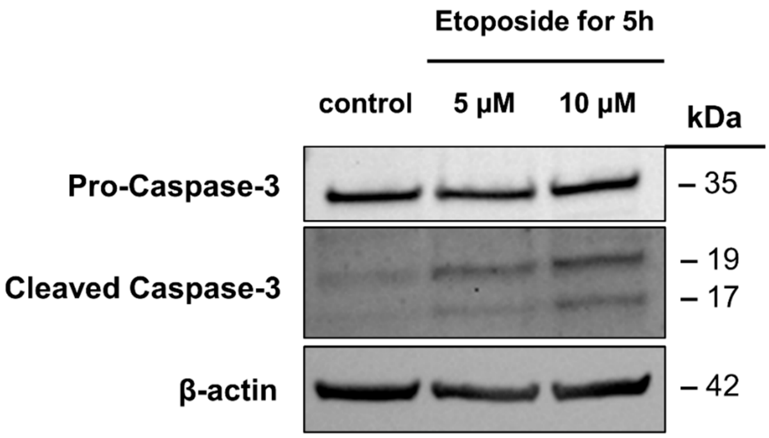

Figure S4. Apoptosis positive control obtained by treating MSCs with final concentration of 5 µM and 10 µM of etoposide for 5h**.** Western blot showing protein level of Pro-Caspase-3 (35 kDa), Cleaved Caspase-3 (19-17 kDa) and β-actin (42 kDa), comparing the untreated control with the cells treated with etoposide.
